# Controlled Drug Release from Biodegradable Polymer Matrix Loaded in Microcontainers Using Hot Punching

**DOI:** 10.3390/pharmaceutics12111050

**Published:** 2020-11-03

**Authors:** Ritika Singh Petersen, Line Hagner Nielsen, Tomas Rindzevicius, Anja Boisen, Stephan Sylvest Keller

**Affiliations:** 1 DNRF and Villum Fonden Center for Intelligent Drug Delivery and Sensing Using Microcontainers and Nanomechanics, IDUN, DTU Health Technology, Technical University of Denmark, 2800 Kgs. Lyngby, Denmark; lihan@dtu.dk (L.H.N.); trin@dtu.dk (T.R.); aboi@dtu.dk (A.B.); suke@dtu.dk (S.S.K.); 2 National Centre of Nano Fabrication and Characterization, DTU Nanolab, Technical University of Denmark, 2800 Kgs. Lyngby, Denmark; 3 Department of Health Technology, DTU Health Tech, Technical University of Denmark, 2800 Kgs. Lynby, Denmark

**Keywords:** biocompatible and biodegradable polymer, hot punching, oral drug delivery, spin coating, controlled release, zero-order

## Abstract

Microcontainers are reservoir-based advanced drug delivery systems (DDS) that have proven to increase the bioavailibity of the small-molecule drugs, targeting of biomolecules, protection of vaccines and improved treatment of Pseudomonas aeruginosa. However, high-throughput loading of these micron-sized devices with drug has been challenging. Hot punching is a new technique that is a fast, simple and single-step process where the microdevices are themselves used as mold to punch biocompatible and biodegradable drug-polymer films, thereby loading the containers. Here, we investigate the effect of hot punching on the drug distribution as well as drug release from the loaded drug-polymer matrices. Zero-order sustained drug release is observed for the model drug Furosemide embedded in biodegradable polymer, Poly-*ε*-caprolactone, which is attributed to the unique spatial distribution of Furosemide during the loading process.

## 1. Introduction

In the past three decades, there has been an emergence of microfabricated drug delivery systems (DDS) in pharmaceutical research. Nielsen et al. [1] describe one such microfabricated oral DDS, named microcontainers. Microcontainers are micro-reservoir-based DDS providing a protective well around the drug and a dissolvable or degradable coating on the open side of the well for unidirectional drug release. It has previously been demonstrated in Mazzoni et al. [2] that the application of microcontainers for the oral delivery of small drug molecules has resulted in higher oral bioavailibity when tested in vivo in rats. Ex vivo perfusion studies in rats illustrated in Mosgaard et al. [3] indicate that the microcontainers sink in the intestinal mucus, leading to a higher absorption rate for the drug. However, so far, the drug has been mostly loaded in its powder form into the microcontainers, as shown in Mosgaard et al. [3] and Abid et al. [4]. Therefore, after the coating on the cavity of the microcontainers dissolves, a fast-burst release of the drug has been observed, as in Nielsen et al. [5]. A more controlled or zero-order release from the microcontainers is desired because controlling the drug release rate has several advantages such as enhancing the therapeutic effect, minimizing the side-effects and decreasing the administration frequency, as stated in Wen and Park [6]. By embedding the drug in a polymer matrix, controlled release of the drug due to the presence of the polymer matrix can be obtained. Therefore, methods such as the crosslinking of drug-loaded hydrogels or the combination of inkjet printing and supercritical impregnation for the loading of reservoirs-based devices, like microcontainers, have been developed. These methods have been reviewed in detail by Fox et al. [7]. Earlier, we have demonstrated in Petersen et al. [8] that a hot punching process can be applied to load the drug-polymer matrix in microcontainer. During this drug loading process, the microcontainers are embossed in a drug-polymer film spin-coated on a deformable layer. Due to the underlying deformable layer, the drug-polymer film is punched along the wall of the microcontainer, thereby filling the microcontainer cavity with drug-polymer matrix. 

In the review by Nicolas et al. [9], it has been stated that biodegradable and biocompatible polymers are being utilized as matrices for controlled release dosage forms. One such polymer that has been studied for controlled drug release is poly-*ε*-caprolactone (PCL). Woodruff and Hutmacher [10] discuss the properties of PCL leading to its extensive application in biomaterials and drug delivery devices. PCL is a man-made polyester with a low glass transition temperature, T_g_, of around −60 °C. The semi-crystallinity of PCL due to its low T_g_ leads to its high permeability, which has been applied for delivery of drugs with low molecular weight. Again, due to the semi-crystalline nature of PCL, it degrades at a slower rate as compared to other biocompatible polymers, such as poly(l-lactide) (PLLA) and poly(lactide *co*-glycolide) (PLGA). Drug release from PCL matrices is therefore often dictated by drug diffusion, which makes PCL suitable for controlled and sustained drug release. However, since the drug release from PCL is mainly driven by diffusion, drug distribution within the polymer matrix influences the drug release rate to a large extent. In previous studies like Wang et al. [11] and Schlesinger et al. [12], the influence of the drug properties and polymer composition on the drug release from PCL microspheres and thin films has been investigated. 

In this paper, we, for the first time, investigate the effects of spin coating and hot punching on the drug crystallinity, distribution and release from PCL matrix, loaded in microcontainers. As a model drug, Furosemide (Furo) was used, which is a loop diuretic. The solubility of Furosemide in water at room temperature is reported to be 18.25 µg/mL. However, the solubility of Furosemide increases with increasing temperature and pH, being 1.9 mg/mL at 37 °C in pH 7.4, as mentioned in Granero et al. [13]. The transient high blood concentration of furosemide after its administration poses some serious side-effects, such as excessive dehydration, pancreatic inflammation, liver damage and hearing loss. Thus, controlled release of furosemide is desirable. For the hot punching process, drug-polymer films with a thickness approximately matching the depth of the empty container reservoir have to be prepared and typically heated to elevated temperatures to achieve loading of the drug-polymer matrices. Therefore, the drug release from spin-coated PCL-Furo films with different thicknesses and processed at different temperatures was evaluated. For this purpose, the spin coating of a homogenous solution of furosemide and PCL was optimized to obtain PCL-Furo films with uniform thicknesses. The solid state of furosemide in the PCL-Furo matrix was investigated using an X-Ray Diffractometer. Next, hot punching was performed and established as a viable method to load PCL-Furo matrices in the microcontainers. The influence of the loading process on the drug distribution and hence the drug release from the PCL matrix was explored using Raman spectroscopy and micro-dissolution techniques.

## 2. Materials and Methods

### 2.1. Materials

Silicon wafers were obtained from Okmetic (Vantaa, Finland) and Polydimethylsiloxane (PDMS) was acquired from Sylgard 184, Dow Corning (Auburn, MI, USA). To prepare PDMS, the PDMS prepolymer is mixed with the curing agent in the ratio of 10:1 *w/w*. Dichloromethane (DCM) (anhydrous >99.8%) and Poly-*ɛ*-caprolactone pellets (average M_w_ ~ 100,000) were purchased from Sigma-Aldrich (Copenhagen, Denmark). Furosemide (≥98% purity) and Phosphate buffer saline (PBS) tablets were procured from Sigma-Aldrich (St. Louis, MO, USA). Milli-Q water was obtained from Merck Millipore (Burlington, MA, USA).

### 2.2. PCL-Furo Spin Coating and Loading of SU-8 Microcontainers with PCL-Furo

**Figure 1:**
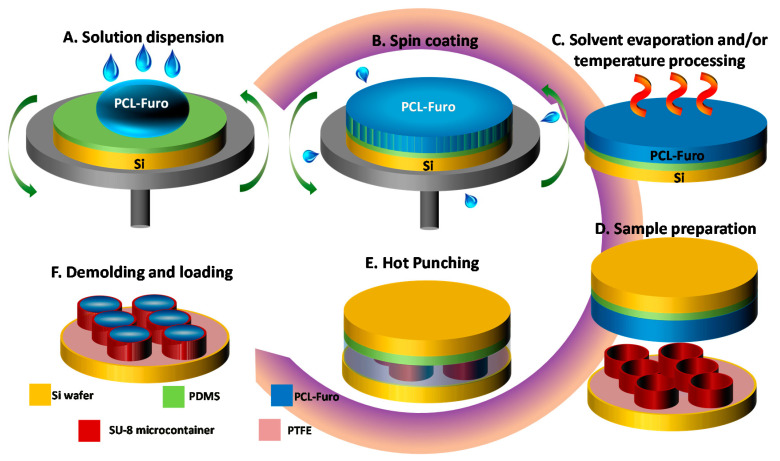
(**A–C**) Spin coating process: (**A**) Poly-ε-caprolactone (PCL)-Furosemide (Furo) solution dispersion on Polydimethylsiloxane (PDMS)-coated Si wafer, (**B**) spin coating at 500 rpm, (**C**) heating of the spin-coated PCL-Furo film. (**D–F**) Hot punching process for loading of spin-coated PCL-Furo films in SU-8 microcontainers: (**D**) sample preparation including the spin coating of a double layer of PCL-Furo solution and stacking of SU-8 microcontainers wafer and PCL-Furo wafer, (**E**) hot punching, (**F**) completed loading of the spin-coated PCL-Furo film in the SU-8 microcontainers after peeling of punched PCL-Furo film between the devices.

For spin coating, first, a homogenous 14.7 *wt*% PCL-Furo (4:1) solution was prepared by mixing 2 g of furosemide (2.9 *wt*%) and 8 g of PCL (11.8 *wt*%) in 20 mL of DCM and 40 mL of acetone. The Si substrate was prepared by spin coating a PDMS layer on a Si wafer. The PDMS solution was spin-coated at 500 rpm speed and at 500 rpm/s acceleration for 60 s. The PDMS layer was hard baked at 90 °C for 30 min on a hotplate, resulting in 80 µm thickness. The polymer-drug solution was dispensed on the PDMS-coated Si wafer for spin coating (Figure 1A). The wafer was accelerated at 500 rpm/s to rotate at 500 rpm for 60 s (Figure 1B). After spin coating, the PCL-Furo films were left overnight for 24 h at room temperature for solvent evaporation. For measuring the effect of temperature, the PCL-Furo films were heated on a hotplate with its temperature stabilized at 40 °C, 65 °C or 100 °C for 30 min (Figure 1C). After that, the films were cooled down slowly at the rate of 2 °C/min to room temperature. The effect of thickness of the PCL-Furo films on the solid-state form of furosemide was studied. Three different thicknesses of the films were obtained, by spin coating multiple layers of PCL-Furo solution: 73 ± 2 µm (3 layers), 42 ± 4 µm (2 layers) and 16 ± 2 µm (1 layer) (Supplementary Materials, Figure S1). All the solid-state characterizations and release studies were performed within 7 days after preparation. The samples were stored in a dark chamber at room temperature with low vacuum to avoid photodegradation of Furosemide and effects of humidity, as prescribed by Asker et al. [14].

Microcontainers made of negative photoresist SU-8 were fabricated on a 4-inch Si wafer using two steps of photolithography, similar to the process described in Nielsen et al. [1]. The SU-8 microcontainers had a height of 100 µm, a diameter of 300 µm and a reservoir with a depth and diameter of 60 µm and 223 µm, respectively. The microcontainers were fabricated on fluorocarbon (FC)-coated Si in order to allow for their final release from the Si substrate. The FC layer was deposited using plasma polymerization.

A double layer of PCL-Furo solution was spin-coated onto the PDMS-coated Si substrate at 500 rpm speed and at 500 rpm/s acceleration for 60 s. The spin-coated wafers with the PCL-Furo films were left in a fumehood overnight to allow complete evaporation of the solvents. After the sample preparation (Figure 1D), the PCL-Furo film was embossed with the wafer containing SU-8 microcontainers as a stamp for 7 min at 65 °C temperature under 1 bar pressure (Figure 1E). At this stage, the PCL-Furo film was punched out from the rest of the film along the walls of the microcontainers. The microcontainers were demolded and the punched drug-polymer matrix was transferred into the reservoirs of the microcontainers (Figure 1F). The film around the punched matrices was finally removed manually. The yield of loading per punching is defined as the number of containers loaded per total number of containers on a Si wafer. The yield is calculated by counting the loaded containers under an optical microscope. The solid-state characterizations and release studies were performed within 7 days after drug-polymer loading.

### 2.3. Scanning Electron Microscopy (SEM) and Profilometry

A TM3030Plus (Hitachi, Tokyo, Japan) tabletop SEM was used for scanning electron microscopy (SEM). The micrographs were obtained at an operating voltage of 15 KV in charge reduction mode using mixed secondary electrons (SE) and back-scattered electron (BSE) detector signal. The thicknesses of the PCL-Furo films were measured using a Dektak XTA stylus profiler (Bruker Karlsruhe, Germany). 

### 2.4. X-ray Powder Diffraction of PCL-Furo Films and Matrices Loaded in Microcontainers

X-Ray powder diffraction (XRPD) measurements were performed on the PCL-Furo films and PCL-Furo loaded in microcontainers using an X′Pert PRO X-ray diffractometer (PANalytical B.V., Almelo, The Netherlands; MPD PW3040/60 XRPD; Cu KR anode; λ = 1.541 Å). The tube voltage and current were set at 45 kV and 40 mA, respectively. A starting angle of 5° 2θ and an end angle of 35° 2θ were employed for the scans. A scan speed of 0.6565° 2θ/min and a step size of 0.01313° 2θ were used. Data was collected using the X′Pert Data Collector software (version 2.2, PANalytical B.V., Almelo, The Netherlands, 2003).

### 2.5. Raman Spectroscopy of PCL-Furo Loaded into the Microcontainers

A DXR Raman microscope (Thermo Fisher Scientific Inc., Waltham, MA, USA) was used to obtain the Raman spectra from the PCL-Furo formulations loaded into SU-8 microcontainers. A single-mode diode laser (780 nm) with the range of 350–2400 cm^–1^ was used to collect the spectra. During the collection, 24 mW laser power was applied that gave an approximated resolution of 2.4–4.4 cm^−1^. An objective of 10x was used to focus on the loaded PCL-Furo and an exposure time of 20 s and five scans were used to collect the data from the surface in focus. The spectra were collected on at least five microcontainers randomly located on one diced chip. The Si wafer with loaded microcontainers was diced into chips containing 25 × 25 microcontainers using a diamond cutter along the crystalline Si planes. For each microcontainer on the chip, three different positions were selected for performing Raman: one spectrum in the center and two spectra near the walls of the microcontainers. 

### 2.6. In Vitro Release of Furosemide from the Spin-Coated PCL films and PCL Matrix Loaded in Microcontainers

The PCL-Furo film was peeled from the Si substrate and cut into individual 1 × 1 cm square samples using a laser. With the help of carbon tape, the cut PCL-Furo films were attached to cylindrical magnetic stirring bars. The magnetic bars with the films were positioned in the bottom of glass vials and covered with 10 mL PBS (pH 7.4) solution. Experiments are performed at 37 °C, while the magnets were stirred at 100 rpm. µDiss (microdissolution) profiler (Pion, East Sussex, UK) was used to record the drug release by using UV probes of path length = 1 mm. A similar set-up was used for PCL-Furo-loaded microcontainers. The chip with 625 microcontainers was attached to the magnets and the drug release was measured with in situ UV probes of 5 mm pathlength. Each channel was calibrated with its own standard curve prior to the release experiments. A fixed volume of furosemide stock solution in PBS buffer was added to 10 mL of PBS. The concentration of the furosemide in PBS media was defined for the UV absorbance spectra. Once the standard curve for furosemide was achieved, in situ measurement of UV absorbance by furosemide was obtained. The samples were scanned every 30 s for 5–7 h by the in situ UV probes. The release tests were conducted for 5–7 h because this duration is longer than the average transit time for drug in pellet or capsule form through the small intestine, as mentioned in Davis et al. [15]. The concentration of furosemide released in the media was determined by analyzing the data at a wavelength range of 275–285 nm. The release experiments are expressed as normalized (to the maximum drug released) drug release over time, and the experiments were performed in 6 replicates from 3 independent productions. 

## 3. Results and Discussion

### 3.1. Effect of Film Thickness and Temperature on the Solid State and Release of Furosemide

For optimal loading with hot punching, it is important to prepare uniformly thick drug-polymer films and to ensure that the elevated processing temperature is not harmful to the embedded drug. Therefore, spin coating of drug-polymer films was optimized, and a study was performed to simulate the effect of processing temperature on the release of furosemide from PCL-Furo films. Three temperatures were chosen for 30 min of annealing after spin coating: 40 °C, which is below the melting point of PCL (T_m_ = 65 °C), 65 °C, being approximately the melting temperature of PCL, and 100 °C, which is above T_m_ for PCL but still below the decomposition temperature of furosemide (220 °C). The melting point of PCL is described in Woodruff and Hutmacher [10], while the decomposition temperature of furosemide is noted in Beyers et al. [16]. Similar temperatures for Tm of PCL and decomposition temperature of Furosemide were observed by the differential scanning calorimetric curves obtained for Furosemide powder, PCL films and PCL-Furo films as shown in Supplementary Materials, Figure S2.

**Figure 2:**
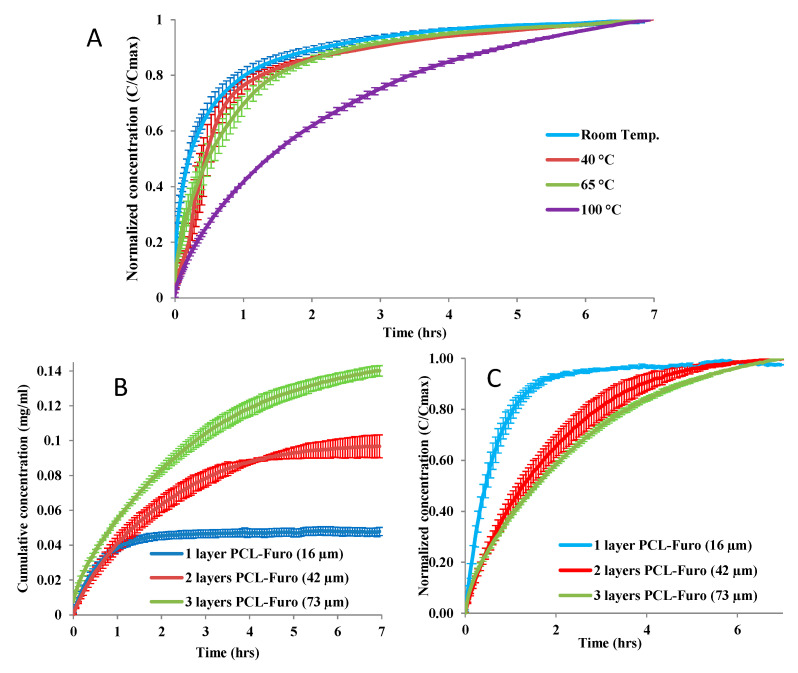
Release profiles of furosemide in pH 7.4 Phosphate Buffer Saline (PBS) showing (**A**) slower furosemide release from 16 µm thick PCL-Furo films annealed at various temperatures, (**B**) higher amount of released furosemide from thicker films and (**C**) a slower furosemide release with increasing film thickness.

The release studies (Figure 2A) showed that the release rate was lower for higher temperature, significantly declining for the film heated at 100 °C. As expected, the thicker films release more furosemide as they contain larger amounts of the drug (Figure 2B). Considering the release rate, one-layered PCL-Furo films showed fast release with almost 80% of the drug released within the first 60 min, while the release from two-layered and three-layered films was much slower (Figure 2C). PCL is a hydrophobic polymer with very slow degradation time. Furthermore, no signs of swelling or erosion in aqueous medium were observed for a duration of 5 h. This implies that the release of furosemide occurred mainly due to diffusion through the matrix. Thus, it is expected that the furosemide release from the spin-coated PCL matrix should represent time-dependent Fickian release profiles, such as the ones in Figure 2. Brophy and Deasy [17] have investigated in depth the diffusion-based drug release from polymer matrices for up to 5–8 h of dissolution time and observed similar time-dependent release profiles.

**Figure 3:**
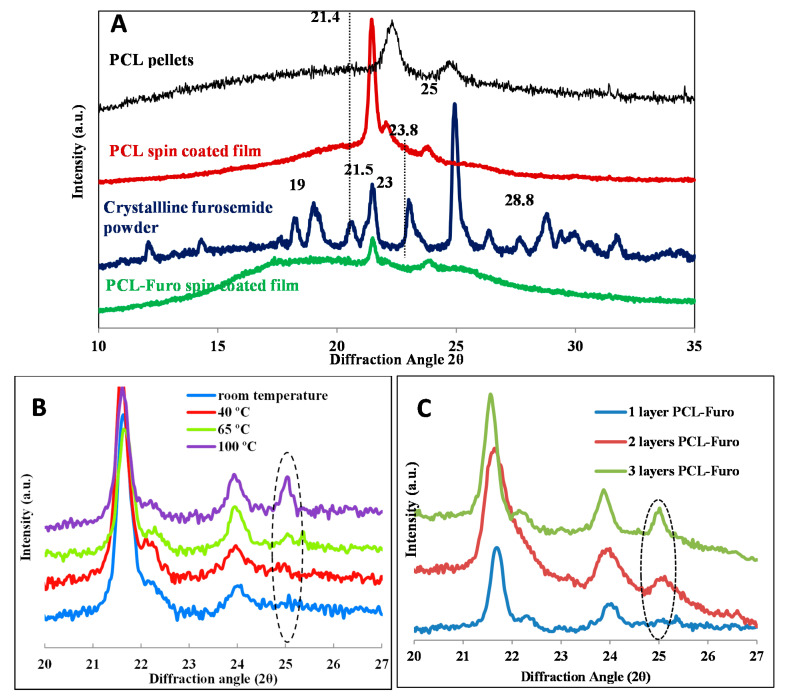
X-ray powder diffraction (XRPD) diffractograms: (**A**) PCL pellets, PCL film (film thickness), powdered crystalline furosemide and spin-coated PCL-Furo film, (**B**) showing increasing crystallinity with increasing temperature of the spin-coated PCL-Furo films and (**C**) showing increasing crystallinity with increasing thickness of the PCL-Furo films.

To further investigate and validate the release profiles, X-Ray Powder Diffraction (XRPD) was performed. In Figure 3A, as-purchased crystalline furosemide powder shows five characteristic peaks at 19°, 21.5°, 23°, 25° and 28.8° 2θ angles, similar to ones seen in Matsuda and Tatsumi [18], confirming that the drug indeed is in its crystalline form I. The most distinct peak was found at 25°. The spin-coated PCL films displayed two main peaks at 21.4° and 23.8° 2θ angles. These match with the peaks reported in Bittiger et al. [19] for PCL films produced by spin coating of PCL solution in DCM. It should be noted that there is a significant sharpening and shift of the peaks for the PCL film as compared to the peaks for the PCL pellets. This is due to the presence of residual stress in the spin-coated PCL film, as elaborated in the notes from Hutchinson [20]. The PCL-Furo film in Figure 3A shows two peaks matching the ones for PCL. Absence of furosemide peaks indicates that furosemide is present in an amorphous form in the film after the spin coating of the drug-polymer matrix. The reason for the amorphous state of furosemide is probably the fast solvent evaporation during spin coating, which basically quenches furosemide in the polymer matrix, as also observed by Van Eerdenbrugh and Taylor [21]. It has previously been shown by Nielsen et al. [22] that a stable amorphous furosemide-polymer solid dispersion can be obtained by spray-drying, increasing the solubility and dissolution rate of furosemide. Figure 3B demonstrates that the main furosemide peak at around 25° increases in its intensity and becomes sharper as the annealing temperature is increased. This implies that there is an increase in the crystallinity of furosemide at higher temperature, which explains the slower drug release for these samples reported in Figure 2. 

Figure 3C illustrates that the furosemide peaks were sharper and are shifted to higher 2θ values for thicker films. This indicates that the lattice parameter decreased, which can be correlated with increased crystallinity of the furosemide in the film. 

Based on these results, the parameters for successful loading of PCL-Furo matrices in SU-8 microcontainers can be extrapolated. Since the heating of the films at 65 °C does not affect the release as drastically as heating at 100 °C and is above the melting point of PCL, loading of the matrices was performed at 65 °C. Each microcontainer has a reservoir with an inner diameter of 223 ± 3 μm and a depth of 60 ± 2 µm. The double layer of PCL-Furo films with a thickness of approximately 42 µm enabled both almost complete filling of the empty volume of SU-8 microcontainers as well as high yield of the loading process. Thicker PCL-Furo films can be loaded but that would imply longer processing time, while at the same time, leading to higher crystallinity of furosemide.

### 3.2. Optimization of Loading of SU-8 Microcontainers

**Figure 4:**
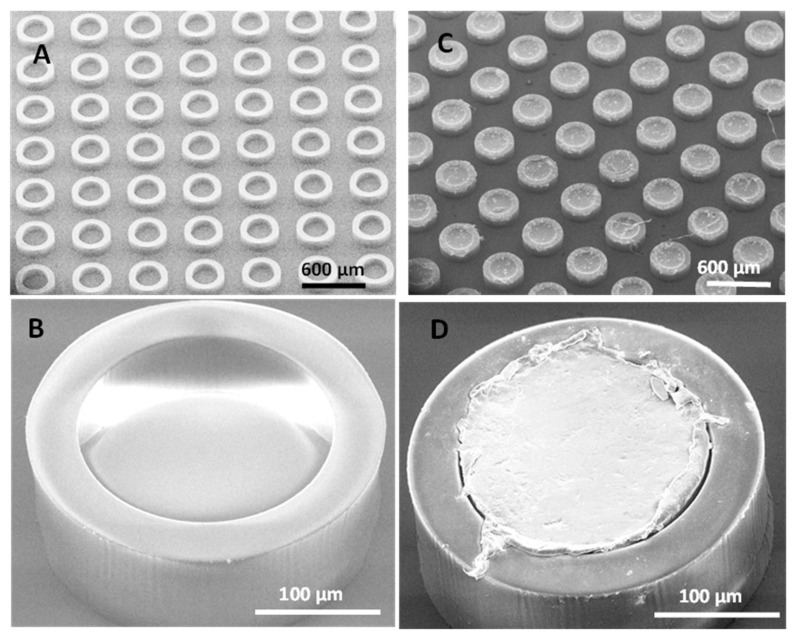
SEM micrographs of: (**A**,**B**) empty SU-8 microcontainers, and (**C**,**D**) high-throughput loading of SU-8 microcontainers with PCL-Furo matrix using optimized process parameters.

The process of hot punching was optimized in order to load the SU-8 microcontainers with PCL-Furo matrices. Figure 4A,B show empty SU-8 microcontainers, while Figure 4C,D show SU-8 microcontainers loaded with PCL-Furo using optimized parameters. The optimized parameters for hot punching of PCL-Furo films into SU-8 microcontainers were a temperature of 65 °C with a pressure of 1 bar applied for 7 min and cooling to 30 °C at the rate of 10 °C/min Higher pressure or faster ramping rate were not ideal for successful loading of microcontainers (Supplementary Materials, Figure S3). It was impossible to punch PCL below its melting temperature due to its semi-crystallinity and PCL is only deformed by embossing the PCL-Furo film around its melting temperature. The transfer of the drug-polymer matrix occurs due to the higher work of adhesion between PCL and the SU-8 container mold compared to the work of adhesion between PCL and the hydrophobic PDMS layer on the Si carrier substrate. In all the experiments, a high yield of loading (>90%) of containers with hot punching was observed (Supplementary Materials, Figure S4). However, as can be noted in the SEM micrograph, there is a lot of empty space between the containers. The film that is removed between the microcontainers can be either reduced by a modified microcontainer design or reused for further loading. XRPD measurement of the Furosemide in PCL loaded microcontainers show a small Furosemide peak indicating that after loading Furosemide is in semi-crystalline state as also observed for the spin-coated PCL-Furo films annealed at 65 °C (Supplementary Materials, Figure S5).

### 3.3. Furosemide Release from the PCL Matrix Loaded in Microcontainers

**Figure 5:**
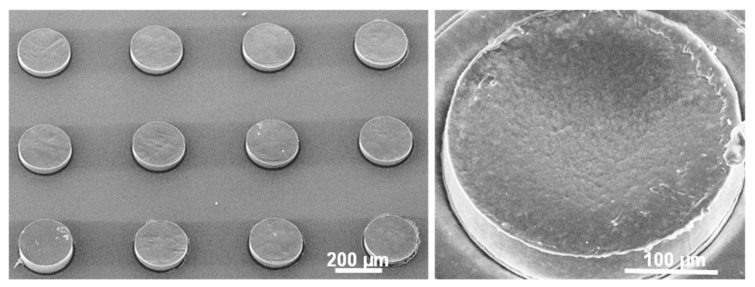
SEM micrograph of PCL-Furo micro-disks prepared by hot punching of PCL-Furo film.

In order to evaluate the effect of physical confinement of the drug on the release profile from the PCL-Furo-loaded SU-8 microcontainers, PCL-Furo micro-disks with the same dimensions as PCL-Furo loaded in microcontainers were used as control. These micro-disks were prepared similar to the drug polymer matrix loaded in microcontainers. However, an ozone plasma treatment of PDMS layer for 20 min before spin coating and hot punching, similar to the one performed in Petersen et al. [23], was introduced, changing the surface energy of PDMS from 6 to 72 mN/m^−1^. Due to the higher surface energy of the treated PDMS layer, the punched PCL-Furo matrix remains attached to the underlying PDMS layer after the demolding of the SU-8 microcontainers (Supplementary Materials, Figure S6), forming PCL-Furo micro-disks, as shown in Figure 5.

The total amount of Furosemide in the 25 × 25 array of SU-8 microcontainers was approximately 200 ± 5 µg. These numbers were obtained by measuring the weight of the chip before and after loading. The total amount of drug released from the loaded SU-8 microcontainers after 5 h was approximately 150 µg, which corresponds to 75% of the drug. This implies that the drug release from the PCL matrix was slow. The reasons for this slow release of furosemide from the SU-8 microcontainers can be manifold, including hydrophobicity of PCL, furosemide and SU-8, and impermeability of SU-8, limiting the drug release to the open side of the reservoir.

**Figure 6:**
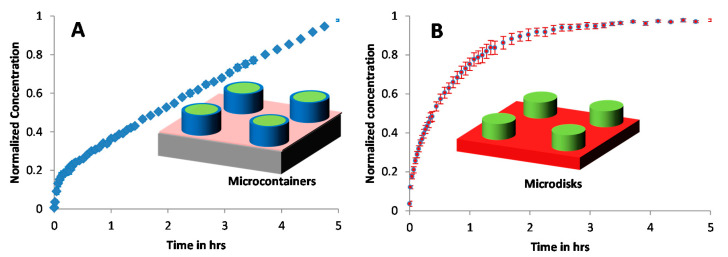
Release of Furosemide from (**A**) PCL-Furo matrix loaded into SU-8 microcontainers, and (**B**) PCL-Furo micro-disks. The release was performed in 10 mM phosphate buffer at pH 7.4.

Figure 6A,B shows the drug release profiles for the PCL-Furo loaded in SU-8 microcontainers and from the PCL-Furo micro-disks where the drug concentrations are normalized with respect to the maximum drug concentration after 5 h of release. It was observed that the furosemide release from the PCL matrix loaded into SU-8 microcontainers showed zero-order kinetics during 5 h of dissolution (Figure 6A) after an initial burst release of around 12% in the first 5–10 min. The initial burst release from a monolithic and reservoir-based drug delivery device could be attributed to the migration of drug molecules to the surface of the device during storage (Supplementary Materials, Figure S7). Huang and Brazel [24] discuss in detail such burst release phenomenon accrediting it to the uneven drug distribution in a matrix-controlled DDS due to diffusion and migration of drug during drying and storage processes. Migration of furosemide in PCL is possible due to the small size and hydrophobicity of the furosemide molecule and the semi-crystalline nature of PCL at room temperature. In Schlesinger et al., drug release from PCL thin films is investigated, where it is reported that small molecules diffuse faster through the PCL film. Further, it is concluded that for the reservoir system, like microcontainers, the transport of hydrophobic drug with larger partition coefficient and lower solubility is faster into the PCL. 

In contrast to the furosemide release from the PCL-loaded microcontainer, the drug release from the micro-disks follows a time-dependent Fickian release profile, similar to the drug release from the spin-coated films (Figure 2). The drug release from polymer matrices is largely influenced by three factors: (1) diffusion coefficient, (2) surface area and (3) concentration gradient of the drug. The surface area and the diffusion coefficient are equivalent for the loaded PCL-Furo matrices and the micro-disks. In our approximate theoretical modelling of the drug release from microcontainers, the Furosemide release from PCL-Furo loaded microcontainers was predicted to be similar to the drug release from microdisks (Supplementary Materials, Figure S8). Therefore, it is hypothesized that the difference between the drug release profiles originates from a non-uniform drug distribution of the drug in the polymer matrix, resulting in a drug concentration gradient. To further investigate the evolution of drug distribution during the loading process using hot punching, Raman spectroscopy was applied.

### 3.4. Drug Distribution in PCL-Furo Matrix Loaded into Microcontainers

**Figure 7:**
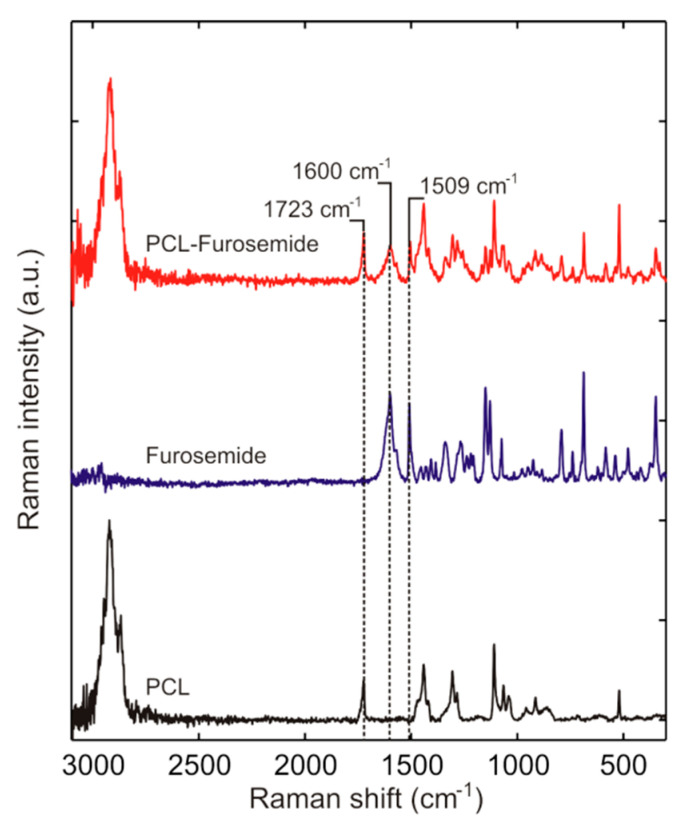
Raman spectra of PCL-Furo matrix loaded into microcontainers as compared to the reference spectra of crystalline Furosemide powder and spin-coated PCL film.

In order to understand the zero-order controlled release of furosemide from the microcontainers, Raman spectroscopy was used to analyze the distribution of drug embedded in the PCL matrix loaded into the microcontainers. In Figure 7, a reference spectrum of furosemide was measured where two distinct furosemide peaks at 1600 and 1509 cm^−1^ can be observed, confirming that furosemide powder (without processing) is in polymorph I form (the most stable). The peaks depicted here match with the furosemide peaks mentioned in Matsuda et al. [25]. The main peak of PCL in the Raman spectrum to which the furosemide peaks were compared to was at 1723 cm^−1^. Kister et al. [26] assign this PCL peak to *ν*C=O stretching mode. As can be seen in Figure 7, the main peaks of furosemide are present in the PCL-Furo matrix loaded in SU-8 microcontainer, showing that embossing at 65 °C temperature and 1 bar pressure did not modify the drug. 

**Figure 8:**
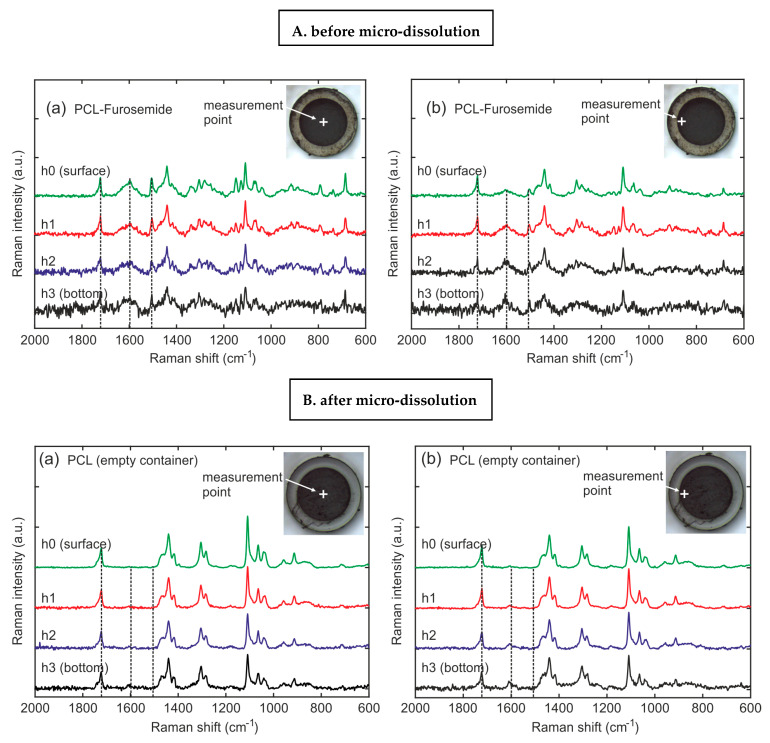
(**A**) PCL-Furo-loaded microcontainers before release, and (**B**) PCL-Furo loaded into the microcontainers after 5 h of release, measured in (a) the center and (b) at the edge of the microcontainer reservoir at four different depths (h0 = surface, h1 = 15 µm, h2 = 30 µm, h3 = bottom).

For investigating the drug distribution, PCL-Furo-loaded microcontainers were characterized in the center and at the edge of the microcontainer reservoir at four different heights from the bottom of the container (Figure 8A(a,b)). Each height difference was 10–15 µm, which was above the depth (Z) spatial resolution. For the qualitative estimation of the drug concentration in the PCL matrix, the ratio of the PCL peak at 1723 cm^−1^ and the furosemide peak at 1600 cm^−1^ were examined. From Figure 8A, it can be seen that the drug concentration is constant with the depth of the reservoir at the center of the matrix. In comparison, the top layers at the edge of the matrix close to the walls of the SU-8 microcontainer reservoir display a lower concentration of furosemide than that at the center and the bottom. Thus, the total drug load increases with the depth of the reservoir of the microcontainers. This heterogeneous drug distribution is accredited to the polymer-drug flow during the hot punching process. Heyderman et al. [27] investigated the flow behavior of polymers during embossing, where the filling of the cavities can be described as a two-steps process. Firstly, due to the capillary forces, the viscoeleastic polymer lying near the borders of the cavity of the stamp rise along the cavity walls. Subsequently, the polymer lying at the center of the cavity, pushed by the rising polymer at the sides, starts filling the cavity. Since hot punching is basically hot embossing with mechanical punching of the polymer film at the end of the process, the polymer flow during hot punching can be explained in the same way as during hot embossing (Supplementary Materials, Figure S9). 

Figure 8B(a,b) show the Raman spectra of PCL-Furo-loaded microcontainers after 5 h of release measured in the center and at the edge of the microcontainer reservoir at four different depths. The overall decrease in the intensity of furosemide peaks after micro-dissolution compared to the peaks before demonstrates that the drug was released in the media. At the same time, the Raman spectra depict that after 5 h of release, there was a higher amount of drug remaining at the bottom of the reservoir than at the top surface. 

## 4. Conclusions

Here, the effect of the hot punching loading process on the drug release from a polymer matrix loaded in microcontainers was studied. The spin coating of optimized homogenous solution of furosemide and PCL provides uniformly thick PCL-Furo films. Furosemide embedded in spin-coated PCL film was found to be amorphous due to the ultrafast quenching of the drug. Spin coating is a viable method for depositing drug-polymer films, where processing parameters influence the crystallinity and morphology of the drug and polymer, and consequently, the drug release from the polymer matrix. These findings could have larger implications for buccal and sublingual drug delivery where multi-layered film systems are applied. 

PCL-Furo matrices were loaded into SU-8 microcontainers using hot punching with >90% yield, at 65 °C and 1 bar pressure. It was demonstrated that the release of furosemide from the PCL matrix provides zero-order controlled release in a 10 mM phosphate buffer at pH 7.4. From the investigation of the drug distribution, it was concluded that loading a drug into microdevices such as microcontainers using hot punching leads to a unique drug concentration gradient in the microcontainers, leading to the zero-order kinetics. In the future, other drug-polymer matrices can be loaded in microcontainers to modulate the release profile and release rate of drugs. In this study, the loading was done in microcontainers fabricated with the prototype material SU-8. In future, this method will be evaluated for biocompatible and biodegradable microcontainers.

## Supplementary Materials

The following are available online at https://www.mdpi.com/1999-4923/12/11/1050/s1, Figure S1. Spin curve for 1-layer PCL –Furo on PDMS coated Si wafer, Figure S2. DSC data of Furosemide powder, PCL and PCL-Furo films, Figure S3. SEM micrograph of failed attempts of loading PCL-Furo in microcontainers, Figure S4. X-Ray tomography showing the filling of SU-8 microcontainers with PCL-Furo matrices, Figure S5. Reference XRPD diffractograms of PCL pellet (purple), PCL loaded in SU-8 microcontainers (orange), PCL film (green), crystalline Furosemide powder (blue) and empty SU-8 microcontainers (black) as compared to the diffractogram of PCL-Furo matrix loaded in microcontainers (red), Figure S6. Hot punching process of loading PCL-Furo matrix in SU-8 microcontainer and fabrication of PCL-Furo microparticles, Figure S7. Raman mapping of the PCL-Furo patch in XZ direction: top (Furosemide peak map), bottom (PCL peak map), Figure S8. Diagram of the drug dissolved from polymer matrix loaded in microcontainer, Figure S9. (a) Ideal distribution of Furo in PCL-Furo film just after spin coating, (b) migration of Furo to the surface of PCL-Furo film due to evaporation of solvent during and after spin coating, (c) Polymer flow along the walls of the stamp at the start of hot punching process, (d) The migration of Furo and polymer flow during hot punching leading a non-uniform drug distribution in the PCL-Furo loaded microcontainers.
