# Supplementary material for: Controlled Drug Release from Biodegradable Polymer Matrix Loaded in Microcontainers Using Hot Punching
Source: Pharmaceutics. 2020 Nov 3;12(11):1050. doi: 10.3390/pharmaceutics12111050 (PMC7692970; doi:10.3390/pharmaceutics12111050)
Supplement: Supplementary file 1 [file pharmaceutics-12-01050-s001.pdf]

pharmaceutics

Supplementary Materials: Controlled Drug Release from Biodegradable Polymer Matrix Loaded in Microcontainers using Hot Punching

Ritika Singh Petersen, Line Hagner Nielsen, Tomas Rindzevicius, Anja Boisen and Stephan Sylvest Keller

1. Spin curve for single-layered PCL –Furo on PDMS coated Si wafer

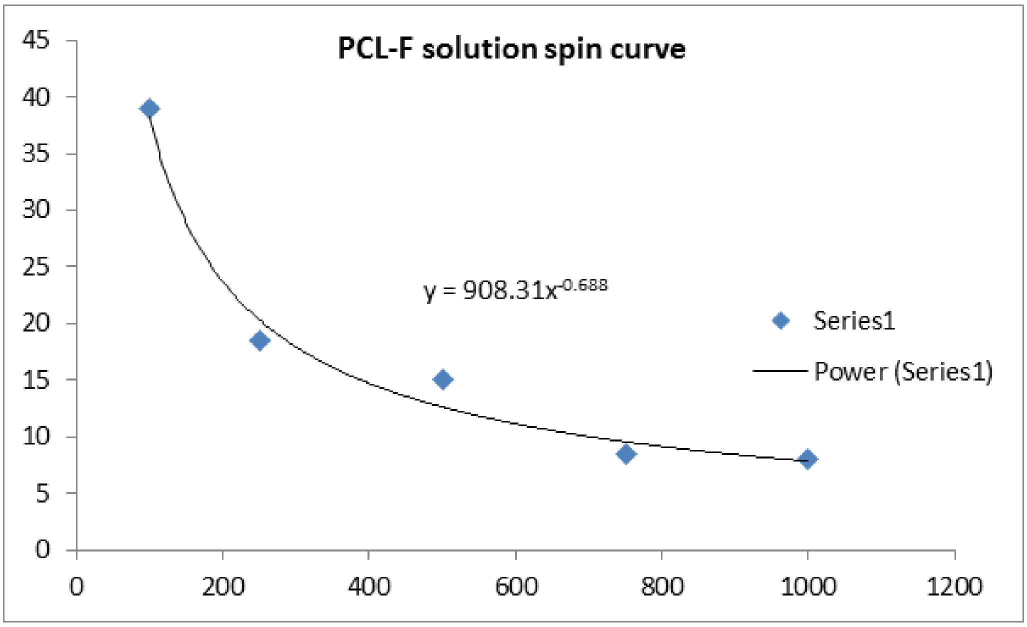

Figure S1. Spin curve for 1-layer PCL –Furo on PDMS coated Si wafer.

2. Differential calorimetry scanning (DSC) data of Furosemide powder, PCL and PCL-Furo films

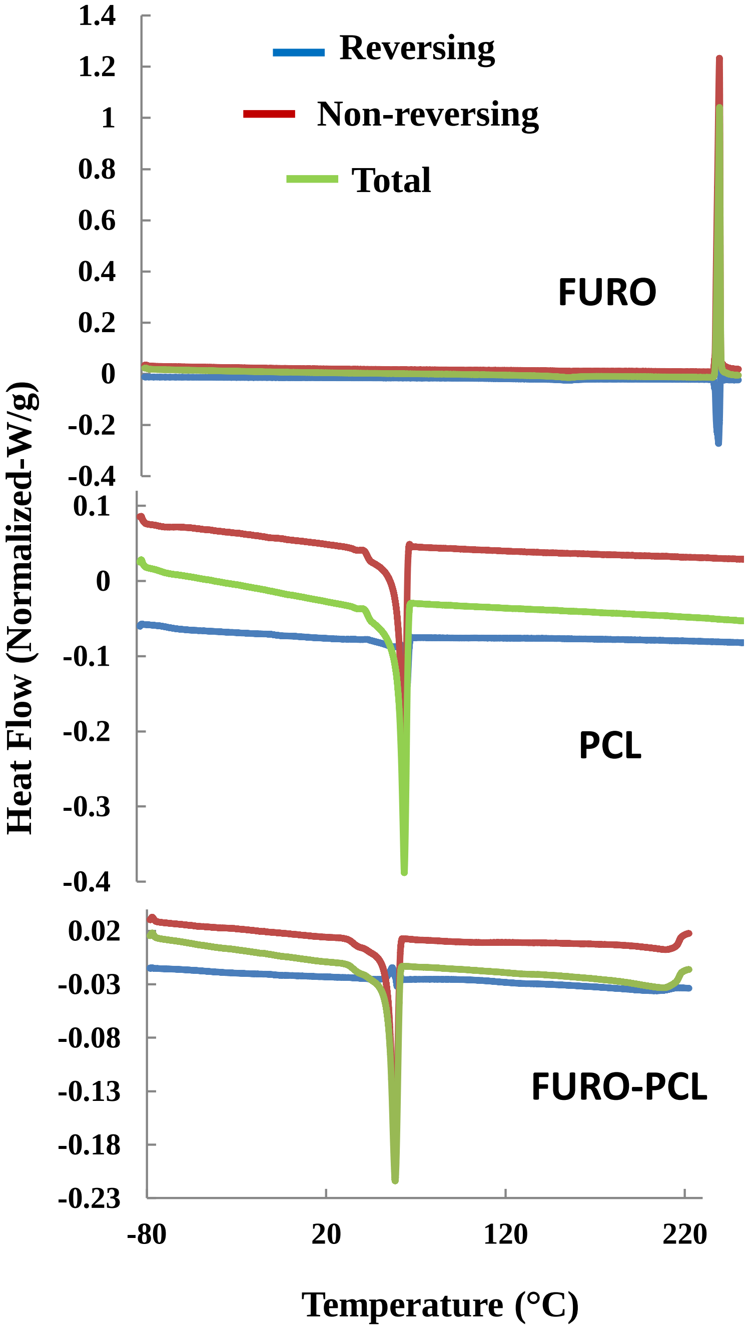

Figure S2. DSC data of Furosemide powder, PCL and PCL-Furo films.

3. SEM micrographs of failed attempts of loading PCL-Furo matrix in microcontainers.

The first (left) image shows the weird motion of PCL-furo that was quenched cooled in liquid nitrogen after hot punching process. The second (right) image shows the destruction of the microcontainers during the loading process under the application of high pressures.

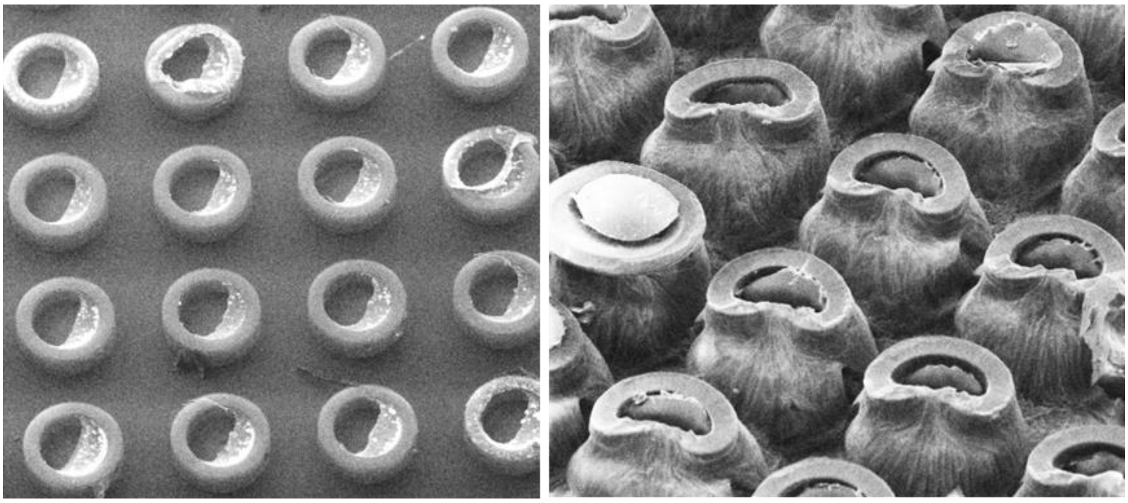

Figure S3. SEM micrograph of failed attempts of loading PCL-Furo in microcontainers.

4. X-Ray tomography of the PCL loaded SU-8 microcontainers

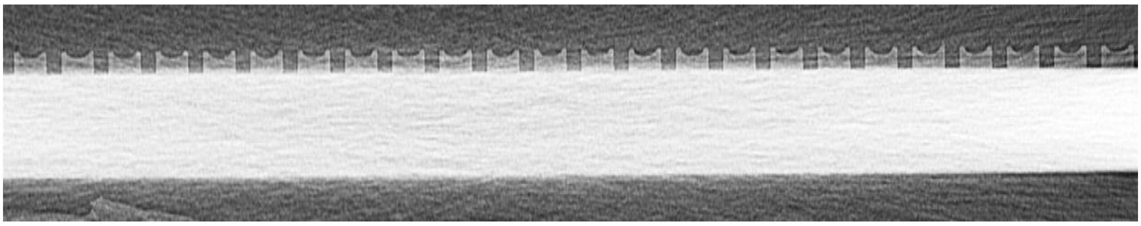

Figure S4. X-Ray tomography showing the filling of SU-8 microcontainers with PCL-Furo matrices.

5. X-Ray powder diffraction (XRPD) of PCL-Furo in containers

The peaks of the PCL matrix loaded into microcontainers shifted to higher angles as compared to the peaks of the PCL films. There is also significant sharpening of the peaks with respect to peaks of PCL pellet and PCL film suggesting that PCL became more crystalline after the hot punching process performed at 65°C. The crystallinity of the polymer matrix affected the crystallinity of Furosemide and hence, the drug release. It has been shown in the literature that the crystals of the polymer matrix can act as the nucleation sites for the drug crystals. In the XRPD of PCL-FURO matrix loaded into microcontainers, a Furosemide peak at 25° 2θ angle can be observed representing crystalline state of Furo. As compared to the spin coated PCL-Furo film without any annealing as in Fig.4.A and B, it can be seen that Furosemide is in amorphous state in the films while it becomes more crystalline after loading in microcontainers due to the heat treatment.

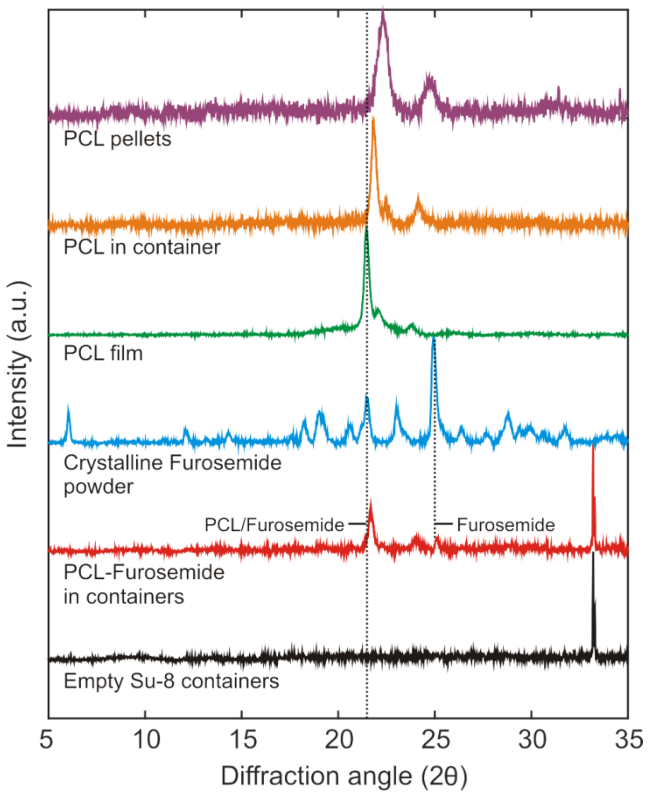

Figure S5. Reference XRPD diffractograms of PCL pellet (purple), PCL loaded in SU-8 microcontainers (orange), PCL film (green), crystalline Furosemide powder (blue) and empty SU-8 microcontainers (black) as compared to the diffractogram of PCL-Furo matrix loaded in microcontainers (red).

6. Fabrication of PCL-Furo microparticles

The fabrication of PCL-Furo microparticles is the same as described as the loading of PCL-Furo in microcontainers, except that here the PDMS layer is treated with UV/Ozone for 20 min, immediately before spin coating of the PCL-Furo solution. The microcontainer chip acts here solely as a stamp for patterning of the PCL-F film. During the demolding of the microcontainers stamp, the punched out PCL-Furo is not transferred into the SU-8 microcontainers but remains on the ozone-treated PDMS film. After peeling of the rest of PCL-F film, the punched out PCL-Furo, which remains on the PDMS film, defines PCL-Furo microparticles (Figure S4.IV).

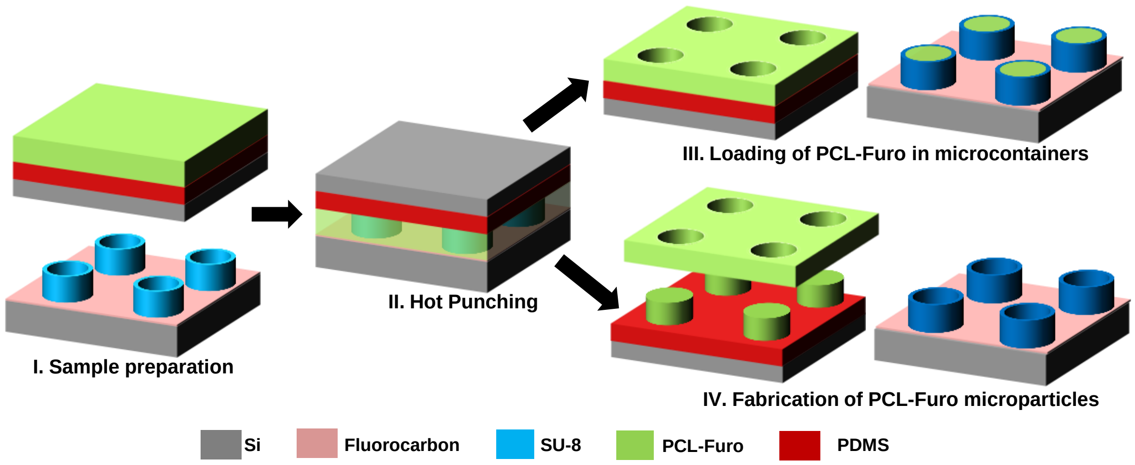

Figure S6. Hot punching process of loading PCL-Furo matrix in SU-8 microcontainer and fabrication of PCL-Furo microparticles.

7. Raman mapping of the PCL-furo patch:

It can be seen from the maps that slightly larger amount of the furosemide sits in the top part of the spin coated PCL-Furo layer.

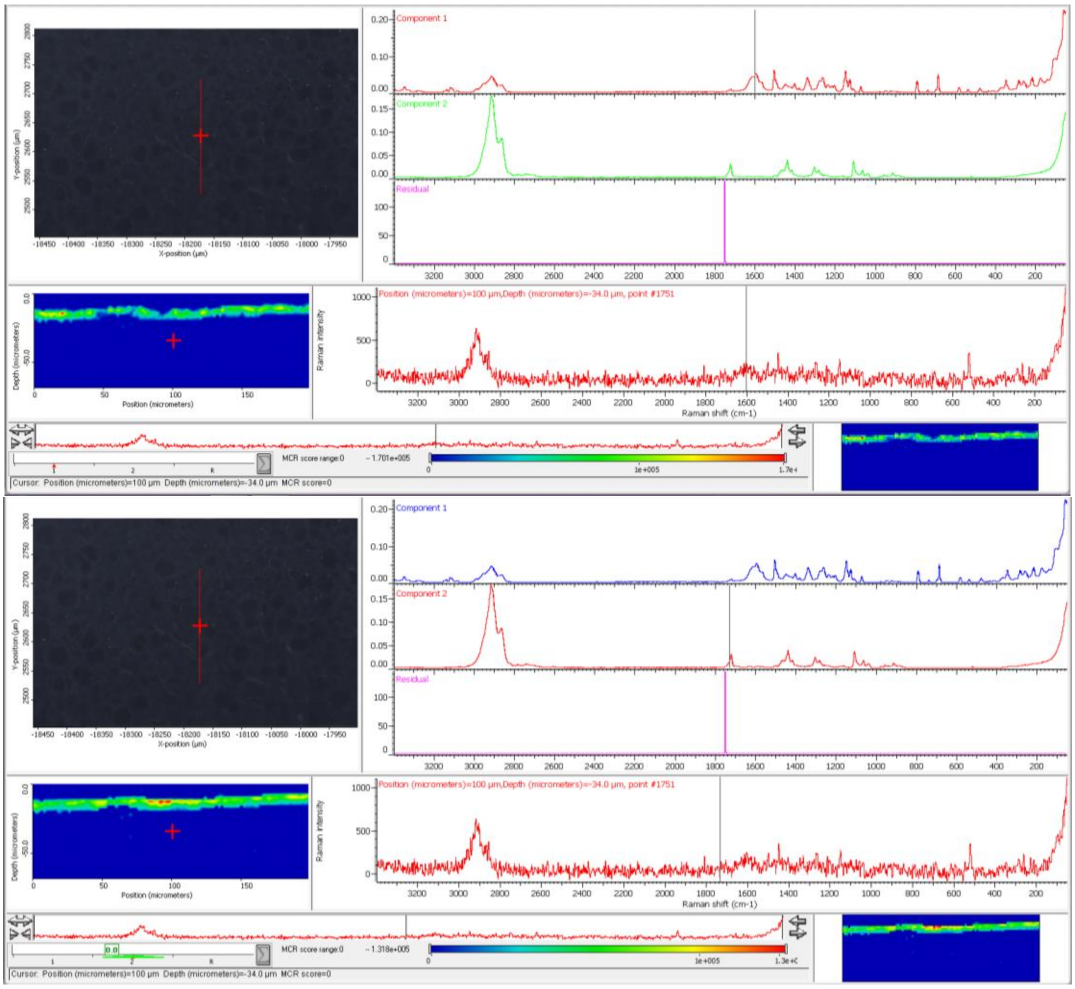

Figure S7. Raman mapping of the PCL-Furo patch in XZ direction: top (Furosemide peak map), bottom (PCL peak map).

8. Theoretical calculations on the dissolution profile of Furosemide (Furo) release from Poly-ε-caprolactone (PCL) matrix loaded in the microcontainers

In the following analysis, drug release from SU-8 microcontainers is modelled. Based on the observations for the spin-coated films, it can be assumed that release of furosemide from PCL is mainly driven by diffusion as the polymer does not erode, degrade or swell in 5–7 hrs. Furthermore, perfect sink conditions, no edge effects and an initial drug concentration in the film much higher than the solubility of the drug, are assumed. The drug release is initiated at the top of the microcontainer and proceeds through the height of the cylindrical reservoir, giving rise to drug release from an infinitesimally thin planar slab. These conditions are very close to the conditions described by Takeru Higuchi in his seminal contribution on the release of a drug from a thin ointment film into the skin. Performing a similar analysis and defining J as the diffusion flux, D the diffusion coefficient of drug, Q(t) the total amount of drug released from the container at time t, r the radius of the reservoir of the container, C(h) the drug concentration (mg/mL) within the matrix at the height h, C_s as drug solubility in the release media which is equal to the concentration of drug at the interface between dissolved (areas where the drug has been released from the matrix) and dispersed (area where the drug is still dispersed but not yet released) regions in the container

reservoir, H as the total height of the reservoir, and C_0 as the initial drug loading per volume of the reservoir of the microcontainer, the release profile can be calculated theoretically:

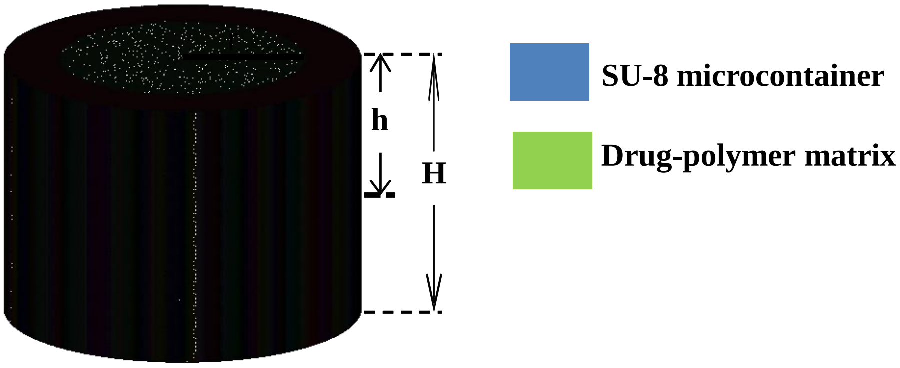

Figure S8. Diagram of the drug dissolved from polymer matrix loaded in microcontainer.

At steady state,

$$J\pi r^2 = \frac{dQ}{dt} \tag{1}$$

Applying Fick's law,

$$J = D\frac{dc}{dh} \tag{2}$$

Using equation 1) and 2), integrating for any height h in the microcontainer and applying boundary conditions: C(h)=C_s,

$$\frac{dQ}{dt} = \frac{\pi r^2 D C_s}{h} \tag{3}$$

The total amount of drug loaded in the microcontainer? is

$$Q(t) = \pi r^2 C_0 h \tag{4}$$

Differentiating 4 with respect to t,

$$\frac{dQ}{dt} = \pi r^2 C_0 \frac{dh}{dt} \tag{5}$$

Equating, 3) and 5) and integrating the equation for time, t

$$h = \sqrt{\frac{2DC_s t}{C_0}} \tag{6}$$

From 3) and 6),

$$\frac{dQ}{dt} = \pi r^2 \sqrt{\frac{DC_0 C_s}{2t}} \tag{7}$$

Thus, from equation 7, it can be extrapolated that the rate of drug release has 1/√t dependence. This means that the release rate declines during the progress of drug release, similar to the drug release from the spin coated PCL-Furo films. However, in practice, such kind of release profile is not observed. This is due to hot punching as loading technique leads to unique drug distribution in the loaded matrix in SU-8 microcontainers. This gives rise to almost zero-order kinetics for drug release.

9. Hot punching leading to unique drug distribution in loaded PCL matrix in SU-8 microcontainers

The unique drug distribution found in the loaded PCL matrix can be accredited to the polymer-drug flow during the hot punching process. Since hot punching is basically hot embossing with mechanical punching of the polymer film at the end of the process, the polymer flow during hot punching can be explained in the same way as during hot embossing. During hot embossing, the filling of the cavities can be described as a two-step process. The filling starts with the flow of the polymer from the borders into the stamp cavity rising up the cavity walls due to capillary forces. Meanwhile, the polymer inside the cavity region prior to embossing does not blend with the polymer flowing in from the borders and instead is pushed by the moving polymer. The consequence of such kind of flow is that if the surface of the spin coated PCL-Furo film is rich with Furo then (Figure 3a) and b)), this drug-rich region is squeezed first along the walls of the microcontainers. Then the central region pushed in the reservoirs (Figure 3c)). Such polymer flow results in the heterogeneous drug distribution, with the Furo-rich region sitting at the bottom of the reservoir, especially near the walls of containers (Figure 3d)). The reason behind Furo-rich PCL film surface can be provided by Huang et.al where it is stated that during the drying and storage of the drug delivery devices, drug has a tendency to migrate to the surface of the device. Thus, when the spin coated film is left overnight to allow the solvents to evaporate, this evaporation and overnight storage leads to a Furo-rich film surface. This in turn led to a non-uniform drug distribution in the microcontainers. Hence, when the amount of available drug is higher in the deeper levels of the reservoir, even though the drug release barrier is increasing, the overall drug release becomes constant over the time. Thus, close to zero order release of Furo is achieved from PCL matrixes loaded into the microcontainers. Since PCL-Furo microparticles are the inverted version of the PCL-Furo matrices loaded in microcontainers, higher amount of drug is present on the top and the sides of the microparticles as compared to deeper areas. This explains the higher tendency for the burst release and the square root time dependent, Fickian release profile for PCL-Furo microparticles (Figure 5). The difference in the release profiles of the PCL-Furo microparticles and PCL-Furo matrices loaded into the microcontainers further validates the evolution of non-uniform distribution of drug obtained during the processing

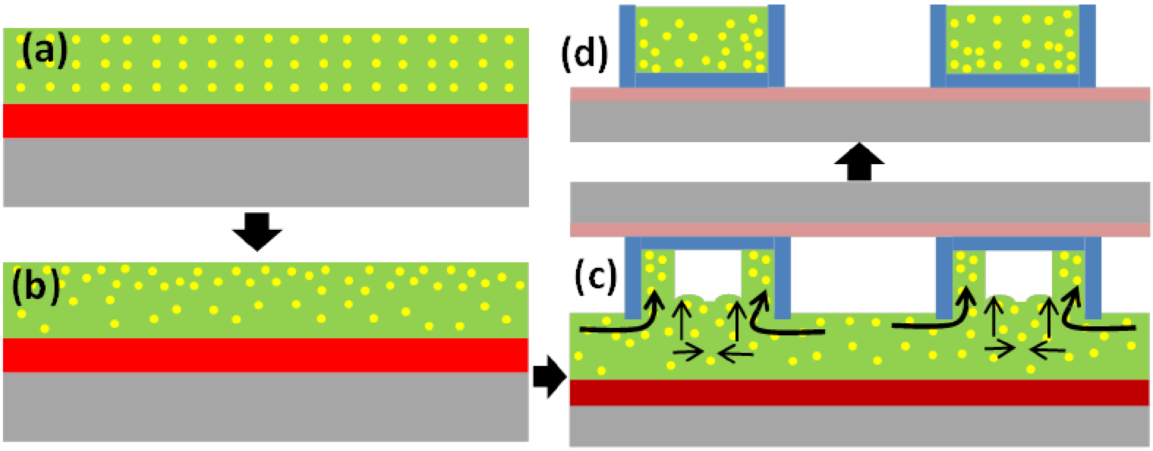

Figure S9. (a) Ideal distribution of Furo in PCL-Furo film just after spin coating, (b) migration of Furo to the surface of PCL-Furo film due to evaporation of solvent during and after spin coating, (c) Polymer flow along the walls of the stamp at the start of hot punching process, (d) The migration of Furo and polymer flow during hot punching leading a non-uniform drug distribution in the PCL-Furo loaded microcontainers.
